# Effect of Different Bleaching Methods on Monomer Release from Aged Microhybrid and Nanohybrid Resin Composites

**DOI:** 10.1155/2023/2773879

**Published:** 2023-05-12

**Authors:** Siavash Abdollahpour, Tarane Estedlal, Nasim Chiniforush, Niyousha Rafeie, Nariman Nikparto, Mahdi Abbasi, Ladan Ranjbar Omrani

**Affiliations:** ^1^Department of Operative Dentistry, School of Dentistry, Tehran University of Medical Sciences, Tehran, Iran; ^2^Department of Surgical Science and Integrated Diagnosis, University of Genoa, Genoa, Italy; ^3^Dental Research Center, Dentistry Research Institute, School of Dentistry, Tehran University of Medical Sciences, Tehran, Iran; ^4^Department of Oral and Maxillofacial Surgery, School of Dentistry, Tehran University of Medical Sciences, Tehran, Iran; ^5^Restorative Dentistry Department, Dental Research Center, Dentistry Research Institute, School of Dentistry, Tehran University of Medical Sciences, Tehran, Iran

## Abstract

**Introduction:**

Recently, the application of laser-assisted bleaching has increased in dental practice. This method might affect the physical and chemical properties of resin composite and its monomer release. This study aimed to evaluate the effect of in-office, at-home, and laser-assisted bleaching on the monomer release (bisphenol A diglycidyl dimethacrylate (BisGMA), triethylene glycol dimethacrylate (TEGDMA), and urethane dimethacrylate (UDMA)) from aged nanohybrid (Grandio, Voco) and microhybrid (Clearfil AP-X Esthetics, Kuraray) resin composites.

**Methods:**

Thirty-two samples of each composite were prepared. The samples underwent aging procedure with UV light at 65°C for 100 hr. The samples were divided into 4 groups: OB: conventional in-office bleaching with Opalescence Boost PF 40% gel; HB: home bleaching with Opalescence PF 15% gel; LB: bleaching with JW Power bleaching gel followed by diode laser application; and C: control group without bleaching. Then, the samples were immersed in a solution containing 75% ethanol + 25% distilled water. The medium was renewed after 8, 16, 24 hr, and 7 days and was analyzed using high-performance liquid chromatography method to evaluate the monomer release. The data were analyzed using Two-way ANOVA and Post hoc Tukey test.

**Results:**

The bleaching method had no effect on TEGDMA and BisGMA release in both composites while it affected UDMA release in nanohybrid composite; UDMA release was significantly higher in LB compared to control and also higher in OB and LB compared to HB. No difference was observed in the microhybrid composite in this regard.

**Conclusion:**

Laser-assisted bleaching did not affect monomer release from microhybrid composite while it increased UDMA release from nanohybrid composite. The bleaching method had no effect on TEGDMA and BisGMA release.

## 1. Introduction

In recent years, the desire for whiter teeth has increased significantly among patients all around the world. Thus, bleaching, as a conservative method for whitening teeth structures has gained noticeable popularity among patients [1]. A variety of bleaching methods and products with different concentrations are available for either in-office or at-home use. According to the literature, in-office bleaching offers several advantages over at-home bleaching, such as better protection of soft tissue, dentist supervision, and faster results [2]. The high concentration of hydrogen peroxide (35%–40%) used during the in-office bleach procedure, produces reactive oxygen molecules and other free radicals which subsequently, break the pigment molecules into molecules that absorb less light [2]. In dental offices, several sources are used to increase the release of free radicals from hydrogen peroxide to improve bleaching efficacy including plasma-arc devices, halogen lamps, light emitting diodes, argon laser, CO_2_ laser, and Erbium YAG laser. Of these sources, diode lasers have become increasingly popular among dentists in recent decades [3–5]. It is believed that lasers improve bleaching efficacy by in-depth activation of hydrogen peroxide. In addition, some laser systems can degrade pigment molecules and result in whiter tooth color [4].

Resin composites are gaining popularity in dental practice due to their aesthetically pleasing appearance as compared to traditional amalgam [6]. Among various types of resin composites, microhybrid and nanohybrid composites are more commonly used for anterior and posterior restorations [7, 8]. However, these restorations are susceptible to discoloration after long-term exposure to food and beverages in the oral cavity [9–11]. Previous studies have shown that bleaching procedures can improve discoloration of resin composite restorations by removing pigments from the composite surface [12–15].

Resin composites are composed of various monomers, including bisphenol A diglycidyl dimethacrylate (BisGMA) and urethane dimethacrylate (UDMA), which are diluted by monomers with lower viscosity, such as triethylene glycol dimethacrylate (TEGDMA) [13]. During polymerization, these monomers react and cross-link to form a polymer network. However, incomplete polymerization can leave some monomers unreacted and trapped in the network [16]. These unreacted monomers can be released from composite restorations [17–20] and cause irritation and allergic reactions in soft tissue as well as inducing bacterial growth [21, 22]. Toxic effects of TEGDMA, UDMA, and BisGMA including cytotoxic, genotoxic, and mutagenic effects have been previously investigated [23]. Issa et al. [24] reported that released monomers decreased mitochondrial activity, and Lefeuvre et al. [25] found that TEGDMA monomer causes mitochondrial damage. Despite these concerns, few previous studies have investigated the effect of conventional bleaching materials on the monomer release from resin composite materials; it has been proposed that bleaching materials including hydrogen peroxide might negatively affect the physical and chemical properties of resin composites [26–29]. According to Polydorou et al. [26], hydrogen peroxide can produce hydroxide (OH^−^), hydroxyl radical (HO·), and hydroperoxyl radical (HOO·), which can react with the carbon–carbon bonds in the resin matrix, resulting in softening and degradation of the composite. This can facilitate the release of unreacted monomers from the composite. Therefore, it is crucial to investigate the type and amount of monomers released from resin composite materials following various bleaching procedures.

Furthermore, with the increasing popularity of laser-assisted bleaching, it is important to evaluate its effect on monomer release from resin composites.

Previous research on the relationship between bleaching and monomer release from resin composite has primarily focused on freshly placed restorations, despite the fact that restorations in the oral cavity are subject to physical and chemical changes over time [30]. Tokay et al. [31], reported that monomer release from resin composite increases after aging, indicating the need to evaluate the effect of bleaching on aged restorations. Hence, the aim of the present study was to evaluate the effect of laser-assisted, conventional in-office bleaching, and at-home bleaching procedures on monomer release from two types of aged resin composites.

## 2. Method and Materials

### 2.1. Sample Preparation

In total, 64 samples were prepared from resin composites (32 from microhybrid (Clearfil AP-X Esthetics, Kuraray) and 32 from nanohybrid composite (Grandio, Voco)) using a 5 mm diameter and 3 mm height plexi mold.  Table 1 summarizes the properties of resin composites used in the present study. A polyester matrix (Kerr Hawe, Switzerland) and a glass plate were positioned on one side of the mold to prevent the formation of an unpolymerized layer on the surface. The molds were filled with resin composite, then another glass plate was placed on the surface of the composite to smooth the surface. Both sides of the mold were cured using a light cure device (Woodpecker LED Curing, Guilin Woodpecker Medical Instrument Co., Guilin, China) with a power intensity of 1,000 mW/cm^2^, each side for 40 s. Then, each sample was polished using a low-speed handpiece and 1,200, 2,400, and 4,000-grit aluminum oxide abrasive disks (Extec, Enfield, CT, USA). Afterward, the samples were transferred to the Xenon test chamber (Alpha LM, Heraeus Kulzer, Hanau, Germany). The Accelerated Artificial Aging procedure was performed at 65°C and 100% humidity for 100 hr.

### 2.2. Bleaching Procedure

After aging, the samples of each composite type were divided into four subgroups and underwent bleaching procedure as follows:

- Control group (C): the samples received no bleaching procedure.
- Office Bleach group (OB): bleaching was performed using 40% Opalescence Boost PF gel (Ultradent Products, Inc, South Jordan, UT, USA). The gel was applied in 2 mm thickness on each sample for 20 min. Then, the gel was removed, and the samples were rinsed completely.
- Home Bleach group (HB): bleaching was performed using 15% Opalescence Boost PF gel (Ultradent Products, Inc, South Jordan, UT, USA). The gel was applied in 2 mm thickness on each sample and the samples were incubated at 37°C and 100% humidity for 56 hr.
- Laser-assisted Bleach group (LB): bleaching was performed using 30% hydrogen peroxide (J White Heydent GmbH, Germany). The gel was applied in 2 mm thickness on each sample and a diode laser (DENZA, GIGAALASER Group, Wuhan, China) was used on each surface three times (each time with 30 s irradiation followed by 1 min interval). The wavelength and output of the device were 810 nm and 1.5 W, respectively. The laser was used in continuous wave mode at a 2 mm distance from each sample surface. Three minutes after the final irradiation, the gel was removed and the samples were rinsed completely.

After bleaching, each sample was fully submerged in a glass tube containing 2 ml of 75% ethanol and 25% distilled water. The tubes were closed, covered with aluminum foil, and stored in a dark room at 25°C. The tubes solutions were changed after 8, 16, 24 hr, and 7 days and high-performance liquid chromatography (HPLC) analysis was carried out each time.

### 2.3. Evaluation of Monomer Release

HPLC analysis was used to measure the amount of released monomer. HPLC device (600 E waters System Controller, Waters, MA, USA) with a Perfect target ODS-3 column (125 × 4 mm, and silica particle size of 5 *μ*) and UV detector at 230 nm wavelength was used for this purpose. Acetonitrile/water in the ratio of 70% : 30% was used as the mobile phase and the flow rate and injection volume were set at 0.8 ml/min and 20 *µ*l, respectively at room temperature which was 20°C. The application was isocratic as the mobile phase mixture was consistent over testing time.

Calibration curves were constructed for each monomer. Pure monomers were used as standards to be utilized in the HPLC system calibration. For this purpose, Pure BisGMA, TEGDMA, and UDMA were used. Standard solutions were made in 75% ethanol and 25% water mixture to obtain the required concentrations. Standard working solutions were prepared separately for each monomer at a concentration of 5, 10, 25, and 50 *μ*g/ml. The amount of monomers in the samples was calculated using standard curves obtained from standard solutions. The identification and quantitative analysis of the monomers were performed using the method described by Barutcigil et al. [32].

### 2.4. Data Analyses

Considering the normal distribution of the data verified by the Kolmogorov–Smirnov test, Two-way ANOVA followed by post hoc test (Tukey HSD) was performed to evaluate the effect of composite type and bleaching method on the monomer release. The significance difference level was considered less than 0.05.

## 3. Results

**Figure 1:**
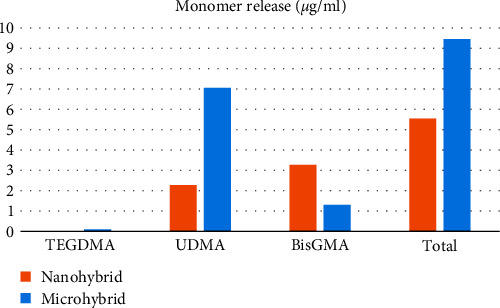
Amount of monomer release in nanohybrid and microhybrid resin composites. Asterisk sign ( ^*∗*^) indicates the significant difference for each type of monomer in comparison between nanohybrid and microhybrid resin composites.

Based on the results of the analyses conducted, it was observed that the total amount of TEGDMA release was not significantly affected by either composite type or bleaching method. On the other hand, for UDMA, the composite type had a significant effect on the amount of monomer release, while the bleaching method did not show a significant effect. Specifically, UDMA release was significantly higher in microhybrid composite than in nanohybrid composite (*P* < 0.001), as depicted in Figure 1. Additionally, an interaction between the composite type and bleaching method was observed for nanocomposite samples. In this case, UDMA release was significantly higher in LB compared to control (*P* = 0.02), while it was also higher in OB and LB compared to HB (*P* = 0.025 and *P* < 0.001, respectively).

In the case of BisGMA monomer, the composite type showed a significant effect on the total amount of monomer release, whereas the bleaching method did not have a statistically significant effect. Specifically, BisGMA release was significantly higher in nanohybrid composite compared to microhybrid composite (*P* < 0.001), as depicted in Figure 1.

Overall, it was observed that the bleaching method had no effect on the amount of TEGDMA and BisGMA release, while it affected the UDMA release in nanocomposite samples. The composite type had a significant effect only on the UDMA and BisGMA.

### 3.1. Microhybrid Resin Composite (Clearfil AP-X Esthetics)

**Figure 2:**
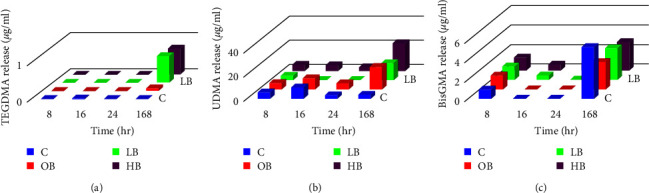
Monomer release after 8, 16, 24, and 168 hr in microhybrid resin composite: (a) TEGDMA release, (b) UDMA release, and (c) BisGMA release.

Regardless of the bleaching method, both UDMA and BisGMA were released from the composite at all time intervals, as illustrated in Figures 2(b) and 2(c). In contrast, TEGDMA was not detected in the solution after 24 hr, and only a small concentration of this monomer was found after 7 days, as shown in Figure 2(a).

In terms of the effect of time on the amount of monomer release, the maximum release of BisGMA and TEGDMA was observed between Day 1 and 7. However, for UDMA, the maximum release occurred in the 8–16 hr interval, after which the release rate decreased notably.

### 3.2. Nanohybrid Resin Composite (Grandio)

**Figure 3:**
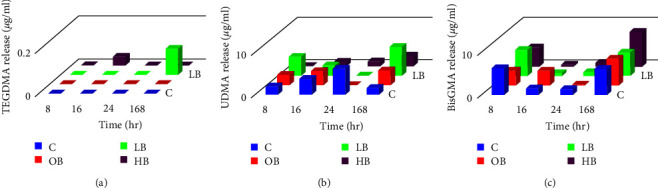
Monomer release after 8, 16, 24, and 168 hr in nanohybrid resin composite: (a) TEGDMA release, (b) UDMA release, and (c) BisGMA release.

The results showed that UDMA and BisGMA were released from the composite at all time intervals, irrespective of the bleaching method, as demonstrated in Figure 3. In contrast, TEGDMA was not detected in the solution even after 7 days.

The release rate of UDMA decreased during the first 24 hr but then increased between Day 1 and 7 compared to the initial 24 hr. On the other hand, for BisGMA, the maximum release was observed within the first 8 hr, followed by a decrease in its release rate during the first 24 hr. However, its release increased again between Day 1 and 7.

## 4. Discussion

In the present study, two types of resin composite including nanohybrid and microhybrid were tested. To simulate the oral conditions, the samples underwent the accelerated aging procedure for 100 hr. The samples were then subjected to a bleaching procedure based on their respective groups. In the LB, diode laser was chosen due to its lower price which makes it a preferable option purchased by many dentists. The applied protocol (three times application, each for 30 s followed by 1 min interval) has been utilized in the previous studies and it is considered a safe protocol for laser-assisted bleaching [2, 33–35]. After bleaching, the bleaching gel was removed from samples using a wet cotton pellet followed by water irrigation to completely remove remained hydrogen peroxide on the samples which simulated the clinical setting as in-office bleaching typically involves rinsing the teeth with water to remove any remaining bleaching agents. Then, the samples were immersed in 75% ethanol + 25% distilled water. According to FDA guidelines, a solution containing 75% ethanol and 25% distilled water can simulate the effects of food and alcoholic beverages used in in vivo conditions [36]. Thus, the amount of monomer release in this medium is comparable to that of the oral cavity [37].

The results obtained from our study showed that UDMA release from nanohybrid composite samples subjected to laser-assisted bleaching was significantly higher compared to other bleaching methods. Moreover, the amount of UDMA release was greater after in-office bleaching compared to control and home bleaching. The increased UDMA release in laser-assisted bleaching and in-office bleaching samples could be attributed to a higher concentration of hydrogen peroxide and titanium dioxide in bleaching gels compared to 15% bleaching gel used in home bleach samples. According to Wiegand et al. [38], the presence of heat and light significantly increases the penetration of hydrogen peroxide into the substrate. Laser acts as an activate energy source and by in-depth activation of hydrogen peroxide, produces more free active radicals and improves bleaching efficacy [34]. In addition, the presence of titanium dioxide in the bleaching gels can enhance the absorption of laser energy, which in turn increases the laser's effects [4]. The combination of higher concentrations of hydrogen peroxide, the presence of titanium dioxide, and the application of laser may promote deeper penetration of hydrogen peroxide into the composite layer. Hydrogen peroxide produces free radicals in deeper layers of composite and these radicals interact with single and double carbon bonds in the composite matrix. This interaction can cause cracks to form in the composite matrix, facilitating the release of unreacted monomers [39].

According to our results, bleaching methods did not affect the TEGDMA and BisGMA release, unlike UDMA release. In contrast to our findings, previous research conducted by Omrani et al. [2] reported a significant increase in BisGMA release following laser-assisted bleaching compared to other groups. They suggested that the more aggressive nature of the laser-assisted bleaching method produced more cracks in the composite matrix, leading to an increase in monomer release. However, we believe that the aging procedure performed in our study may account for the observed difference between the two studies. Aging can weaken the composite matrix to the point that BisGMA release is not significantly different between groups, regardless of the bleaching method.

Durner et al. [27] investigated the effect of bleaching with Opalescence® PF 15% on monomer release from three types of resin composite. Their findings indicated a significant increase in monomer release after conventional bleaching, which is attributed to the production of free radicals by hydrogen peroxide that can break the bonds in the polymer network, leading to increased monomer release. However, in our study, conventional bleaching did not result in increased monomer release compared to the control group. This difference could be explained by the fact that the samples were thoroughly rinsed with water after removing the bleaching gel using a damp cotton pellet, whereas Durner et al. [27] only used a cotton pellet to remove the gel without water. It is possible that wiping alone was not sufficient to remove the bleaching gel completely, and any remaining hydrogen peroxide continued its oxidative activity, resulting in increased monomer release [26].

Moreover, it was observed that the overall monomer release from the microhybrid composite was higher compared to the nanohybrid composite. These findings are contrary to the results of Ferracane [40] who reported a higher release of monomers from nanohybrid composites. Ferracane [40] proposed that the interface between filler and matrix is the most vulnerable site for water absorption and further degradation of composite structure, and hence, nanohybrid composites are more prone to degradation and monomer release due to their smaller filler size and increased filler–matrix interface. However, we believe that the differences between our study and Ferracane's [40] study may be due to the accelerated aging procedure used in our study. According to the literature, nanohybrid composites undergo less degradation after aging compared to microhybrid composites, and their mechanical properties are less affected by aging compared to microhybrid composites [41, 42]. Thus, microhybrid composite experienced more degradation and monomers release after aging. It is important to note that the different study designs and sample preparations between our study and that of Ferracane [40] could also account for the differences in results. Additionally, the type of storage solution and the average storage time are other factors that may have contributed to the different results between the studies.

Our results showed that the amount of BisGMA released was higher in nanohybrid composite compared to microhybrid composite, while microhybrid composite exhibited greater UDMA release. It is important to note that both composites have a similar organic matrix, monomer composition, and filler volume percentage. The only difference between these composites is particle filler size which might contribute to the different amounts of monomer release in these two composites. Furthermore, TEGDMA was not released from nanohybrid composite while microhybrid composite released a small amount of this monomer after 7 days. According to the manufacturer catalog, Grandio nanohybrid composite contains TEGDMA and the release of this monomer has been observed in previous studies [29, 43]. It is probable that during the aging procedure, Grandio composite underwent surface degradation, and due to the lower molecular weight of TEGDMA, this monomer was easily released from the degraded composite surface. The remaining TEGDMA monomer was released from the composite during the bleaching procedure and finally washed away with water after the completion of bleaching.

It is important to note that neither of the resin composites contained UDMA according to the manufacturers, but both of them released UDMA according to the HPLC analysis.

The most important limitation of the present study is that although Accelerated Artificial Aging was used to simulate the oral cavity conditions, it was impossible to thoroughly simulate the oral cavity environment since many other factors exist in the oral cavity that could not be simulated in the laboratory setting.

Further studies might be necessary to evaluate broader types of resin composites with different types of monomers. Different sample preparations might also affect the final result which can be analyzed in future studies.

## 5. Conclusion

Within the limitations of the present study, it can be concluded that the effect of the bleaching method on the amount of monomer release depends on the type of resin composite and monomer type.

## Figures and Tables

**Table 1:** The composition of resin composites used in the present study.

Material	Manufacturer	Type	Monomers of organic matrix	Filler	Particle size range	Filler (%)
CLEARFIL® AP-X esthetics	Kuraray Noritake Dental Inc., Japan	Microhybrid	BisGMA, TEGDMA, and UDMA	Silanated barium glass filler, silanated silica filler	0.02–17 *µ*m	86% (w/w), 71% (v/v)
Grandio®	Voco GmbH., Germany	Nanohybrid	BisGMA, TEGDMA, and UDMA	Glass-ceramic, microfillers, and nanofillers	Glass-ceramic microfillers average particle size: 1 *µ*m, nanofillers range of particle size: 20–50 nm	87% (w/w), 71.4% (v/v)

## Data Availability

The datasets generated during and/or analyzed during the current study are available from the corresponding author upon reasonable request.
